# Ketogenic supplementation enhances KRAS^G12D^ inhibitor efficacy in pancreatic ductal adenocarcinoma

**DOI:** 10.1016/j.canlet.2026.218490

**Published:** 2026-04-08

**Authors:** Loan D. Duong, Julim Lee, Yujing Qin, Nipuni Barupala, Melissa L. Fishel, Xuemin Lu, Jun Wan, Anita A. Turk, Xin Lu

**Affiliations:** a Department of Biological Sciences, University of Notre Dame, Notre Dame, IN, 46556, USA; b Integrated Biomedical Science Graduate Program, University of Notre Dame, Notre Dame, IN, 46556, USA; c Department of Pediatrics, Department of Biochemistry, Molecular Biology, and Pharmacology, and IU Simon Comprehensive Cancer Center, Indiana University School of Medicine, Indianapolis, IN, 46202, USA; d Department of Medical and Molecular Genetics, Center for Computational Biology and Bioinformatics, Indiana University School of Medicine, Indianapolis, IN, 46202, USA; e Department of BioHealth Informatics, Luddy School of Informatics and Computing, Indiana University at Indianapolis, Indianapolis, IN, 46202, USA; f Indiana University Simon Comprehensive Cancer Center, Indiana University School of Medicine, Indianapolis, IN, 46202, USA; g Division of Hematology/Oncology, Department of Medicine, Indiana University School of Medicine, Indianapolis, IN, USA

## 1. Introduction

Pancreatic ductal adenocarcinoma (PDAC) remains one of the most lethal malignancies [1]. KRAS mutations occur in >90% of PDAC, with KRAS^G12D^ as the most frequent allele [1]. The recent development of KRAS^G12D^-selective inhibitors, such as MRTX1133, has shown promise in preclinical and early clinical studies [2]. However, as with most targeted therapies, resistance inevitably emerges, limiting durable tumor control. Understanding mechanisms and developing combinatorial therapy strategies to enhance the efficacy of KRAS inhibition is critical for improving patient outcomes.

Metabolic changes induced by dietary interventions, including ketogenic diets or ketone body supplementation, have attracted interest as adjuvant strategies in cancer therapy [3]. β-hydroxybutyrate (BHB), the principal ketone body produced during ketosis, has been shown to outperform glucose in enhancing CD8^+^ T cell effector function by fueling the tricarboxylic acid cycle and acetyl-CoA production, and by promoting histone acetylation at effector gene loci [4,5]. Given that effector T cells contribute to the efficacy of MRTX1133 in murine PDAC models [6,7], we argue that BHB may augment the effect of the KRAS^G12D^-selective inhibitor.

We recently reported that dietary 1,3-butanediol (1,3-butylene glycol), an effective and convenient method to raise circulating BHB level, demonstrated superior enhancement of immune checkpoint blockade (ICB) in prostate cancer models compared to the ketogenic diet [8]. Here, we evaluated 1,3-butanediol supplement in combination with MRTX1133 in an orthotopic, immunocompetent PDAC model and employed single-cell RNA sequencing (scRNA-seq) and spatial transcriptomics to define changes in the tumor microenvironment (TME).

## 2. Materials and methods

All materials and methods employed to perform this study are described in detail in the Supplementary Data.

## 3. Results and discussion

### 3.1. MRTX1133 mitigates KRAS^G12D^-MAPK signaling and decelerates iKRAS tumor growth

To establish the baseline therapeutic response to KRAS^G12D^ inhibition, we evaluated the effect of MRTX1133 on the doxycycline(dox)-inducible KRAS (iKRAS) mouse PDAC model, specifically a C57BL/6-syngeneic PDAC cell line (AK14838) derived from the *P48-Cre; R26-rtTa-IRES-EGFP*; *tetO-LSL-Kras*^*G12D*/+^; *Trp53*^*L*/+^ genetically engineered mouse model [9]. This system allows dynamic activation or inactivation of KRAS^G12D^: dox-on condition turned on KRAS^G12D^ expression and stimulated MAPK signaling as shown by higher phospho-MEK and phospho-ERK (Fig. S1A). MRTX1133 at 100–300 nM effectively diminished phospho-MEK and phospho-ERK signals on iKRAS cells under dox-on condition *in vitro* (Fig. S1B), an effect sustained for up to 6 h (Fig. S1C).

We labeled the iKRAS cell line with luciferase to enable bioluminescence imaging (BLI) and established orthotopic tumors in C57BL/6 mice under dox-on condition. Extinction of KRAS^G12D^ by doxycycline withdrawal led to rapid tumor eradication and cured nearly half of the mice, whereas MRTX1133 delayed tumor progression, with all mice eventually succumbing to tumor burden (Fig. 1A–B). A similar pattern of limited tumor control by MRTX1133 compared with complete KRASG12D depletion under the dox-off condition was observed in immunodeficient NOD/SCID mice (Fig. S1D–E), indicating that resistance to KRAS^G12D^ inhibition readily emerges regardless of adaptive Immunity. These findings demonstrate that while MRTX1133 provides meaningful therapeutic benefit, KRAS^G12D^ inhibition alone is insufficient for durable tumor control, motivating investigation of combination strategies.

### 3.2. Ketogenic supplementation enhances the therapeutic efficacy of KRAS^G12D^ inhibition

To assess the effects of ketogenic supplementation, we first evaluated β-hydroxybutyrate (BHB) and MRTX1133 in 3D spheroids formed by the iKRAS cell line under dox-on condition. While MRTX1133 reduced spheroid areas and induced apoptosis as indicated by pronounced cleaved caspase 3, BHB did not affect spheroids or apoptosis marker (Fig. 1C–D; Fig. S1F). Therefore, BHB has a negligible effect on the iKRAS model *in vitro*.

Next, we evaluated the single and combination treatment using the iKRAS model in C57BL/6 mice. Two weeks after the pancreas inoculation of iKRAS cells, mice were randomized to receive vehicle, MRTX1133 (30 mg/kg, twice/day), 1,3-butanediol (supplemented in the standard diet), or the combination of MRTX1133 and 1,3-butanediol (Fig. 1E). We confirmed that 1,3-butanediol raised serum BHB levels to 1.0–1.5 mM (Fig. 1F). While 1,3-butanediol supplement alone had little effect on tumor burden, the addition of the 1,3-butanediol to MRTX1133 outperformed MRTX1133 alone, as the combination led to the most pronounced deceleration of tumor progression (Fig. 1G–H) and the best survival outcome, with 50% tumor-free status (Fig. 1I). A similar experiment in NOD/SCID mice also demonstrated the superior efficacy of the combination of MRTX1133 and 1,3-butanediol supplement; however, no complete tumor eradication was observed (Fig. 1J–L; Fig. S1G), suggesting that adaptive immune cells are indispensable for maximal protection by the combination therapy.

### 3.3. Single-cell transcriptomic profiling reveals epithelial state shifts and favorable immune remodeling induced by combined KRAS^G12D^ inhibition and 1,3-butanediol supplement

To understand treatment-induced cellular and molecular changes in the C57BL/6 host, we conducted scRNA-seq using the 10X Genomics workflow on pancreatic tumors harvested 1 week after treatment from the four groups: vehicle, MRTX1133, 1,3-butanediol-supplemented diet (BD), and combination (Combo). This early time point was selected to capture ongoing treatment-induced changes preceding significant diversion of tumor volume across the groups. After quality-control metrics, 13,966 total cells were annotated into major cell populations (Fig. 2A, Fig. S2A). Combo-treated tumors exhibited the lowest proportion of epithelial cancer cells, the lowest proportion of cancer-associated fibroblasts (CAFs), and the highest proportion of T and NK cells (Fig. 2B). The epithelial compartment was further refined into 6 clusters (Fig. 2C), which could be attributed with functional terms based on marker expressions, such as epithelial trait (strongest in cluster 0-Ep and 1-Ep/Ag), epithelial–mesenchymal transition (EMT, strongest in cluster 2-EMT), proliferation (strongest in cluster 3-Pr/Ag and 4-Pr), exocrine pancreas (strongest in cluster 5-Exo), and antigen presentation (strongest in cluster 1-Ep/Ag and 3-Pr/Ag) (Fig. 2D). Pseudotime analysis using Slingshot portrayed a continuous trajectory following clusters 5 → 0→1 → 3→4 → 2, consistent with a process evolving from normal-like (exocrine, antigen-presenting epithelial) towards malignancy (proliferative, EMT) (Fig. 2E). When cluster proportions were analyzed, while BD did not alter portions, MRTX1133 diminished the more malignant clusters 3-Pr/Ag, 4-Pr and 2-EMT while expanding the less malignant clusters 5-Exo and 1-Ep/Ag, with Combo showing abolished cluster 2-EMT (EMT^high^) and most expanded cluster 5-Exo (high in exocrine pancreas) (Fig. 2F). This shift in cluster proportions may reflect transcriptional reprogramming toward exocrine fate, selective elimination of aggressive malignant cell states, transcriptional adaptation to KRAS inhibition, or a combination thereof; we cannot distinguish these possibilities from transcriptomic data alone.

Next, we scrutinized the immune cells. Of the tumor microenvironment populations, the T-NK compartment demonstrated the most dramatic expansion with combination treatment (Fig. S2B). Subclustering this compartment identified more refined lymphocyte subsets, including CD8^+^ T, CD4^+^ T, Tregs, γδ T, NK, NKT cells, and ILC2, based on characteristic marker gene expression (Fig. 2G–H). Compared with Vehicle control, MRTX1133, BD, and Combo resulted in a gradually increasing proportion of CD8^+^ T cells (Fig. 2I), consistent with the beneficial effect of BHB or MRTX1133 on CD8^+^ T cells [4–7]. We subjected the significantly differentially expressed genes (DEGs) in CD8^+^ T cells between Combo and other groups to gene ontology biological process (GO-BP) enrichment analysis. Combo condition positively enriched for pathways representing T cell co-stimulation, TCR signaling, and immune response to tumor cells while negatively enriched for immunosuppressive pathways such as IL-10 and TGFβ/Smad signaling (Fig. 2J–Table S1–S3). Combo-treated CD8^+^ T cells showed notable patterns of gene expression: (i) elevated expression of activation-related genes, such as the ones involved in TCR signaling (*Cd3d, Cd3e, Cd3g, Cd8a, Cd8b1, Zap70, Lat2, Sh2d2a, Malt1*), effector cytotoxicity (*Nkg7, Fasl, Tnfsf10, Ctsw*), co-stimulation (*Cd28, Icos, Cd226, Cd96, Il7r, Il12rb2, Il21r*), transcriptional regulation (*Tbx21/T-bet*), and tissue infiltration (*Itgae/Cd103, Itga1/CD49a, Cxcr6*) (Fig. S2C); (ii) lower expression of genes implicated in exhaustion and suppression (*Nr4a1/Nur77, Eomes, Bach2, Tox, Zeb1*) (Fig. S2D); (iii) higher expression of checkpoint receptors (*Pdcd1, Ctla4, Lag3, Havcr2/Tim3*) (Fig. S2E). The co-occurrence of checkpoint receptor upregulation with a strong effector program and a T-bet^high^ Eomes^low^ transcriptional state is consistent with recently activated, antigen-experienced effector CD8^+^ T cells rather than a terminally exhausted phenotype [10].

Next, we examined myeloid cells, which were reduced most by BD and Combo conditions (Fig. 2B). Based on marker genes, myeloid cells were clustered into neutrophils, monocytes/macrophages, and dendritic cells (Fig. S2F–G). Compared with Vehicle, BD downsized neutrophils and augmented monocytes/macrophages, whereas MRTX1133 altered them in the opposite direction, and Combo reached the average levels (Fig. S2H). For neutrophils, the most substantial myeloid group across the conditions, DEGs between the Combo and MRTX1133 conditions, analyzed through GO-BP over-representation analysis, revealed that the Combo condition was positively enriched for pathways of antigen presentation, inflammatory response, and CD40 signaling, while negatively enriched for neutrophil chemotaxis and glycolysis (Fig. S2I, Table S4–S6). Findings from T cells and myeloid cells indicate that combination treatment provided the favorable remodeling toward an antitumor immune environment.

### 3.4. Spatial transcriptomics reveals combination therapy remodels tumor architecture and immune neighborhoods

Finally, we leveraged spatial transcriptomics (ST) to validate treatment-associated remodeling of tumor architecture. Using 10x Genomics Xenium, we mapped five major cell classes within tumor sections across treatment groups (Fig. 2K; Fig. S2J). Consistent with scRNA-seq, compared with vehicle or monotherapies, Combo-treated tumors exhibited the lowest proportion of epithelial cells and the highest proportion of immunocytes (Fig. 2L). ST also captured an endothelial cell expansion induced by Combo treatment (Fig. 2L), which scRNA-seq missed. Immune cells were further refined to T/NK cells, macrophages, and neutrophils (Fig. S2K–L). Spatial mapping of the cell types in the centroid space revealed that Combo treatments induced the most visible shift in spatial organization, characterized by dispersal of the epithelial compartment and redistribution of T/NK and myeloid cells into regions previously occupied by tumor epithelium (Fig. 2M, Fig. S2M). Concordantly, neighborhood analysis revealed the expected strong self-proximity (diagonal red blocks) for all the cell types and that Combo imposed the strongest rewiring of local cell–cell adjacency (Fig. 2N). The marked depletion of epithelial proximity scores (as low as −2.00) across immune and stromal populations reflects profound spatial reorganization consistent with tumor destruction; residual epithelial cells form isolated self-clustering islands while non-epithelial cells such as T/NK cells occupy the spatially remodeled TME following epithelial loss. These patterns do not indicate immune exclusion from residual epithelium but rather capture the spatial signature of treatment-induced architectural disruption and immune remodeling.

In summary, ketogenic supplementation with 1,3-butanediol potentiates the efficacy of KRAS^G12D^ inhibition in PDAC through immune-dependent tumor control and rapid microenvironment remodeling. Oncogenic KRAS drives anabolic glucose metabolism and immune evasion [9,11,12]. BHB provides a preferred carbon source for effector T cells and CAR-T cells, fueling TCA cycle activity and acetyl-CoA production, promoting oxidative phosphorylation and ATP production, driving histone H3K27 acetylation at effector gene loci, and inducing global transcriptional and epigenetic reprogramming [4,5,13]. In the context of KRAS^G12D^ inhibition, which itself promotes FAS-mediated killing by CD8^+^ T cells [7], BHB may amplify this effect by meeting the heightened metabolic demands of the expanding effector T cells and other antitumor immunocytes. The lack of single-agent activity for BHB *in vitro* and 1,3-butanediol *in vivo* is consistent with BHB being a metabolic modulator rather than a direct cytotoxic agent. Known resistance mechanisms to KRAS inhibitors include bypass signaling, KRAS amplification, secondary KRAS mutations, EMT-mediated resistance [14]. Our data suggest that BHB supplement may overcome or at least delay resistance through both immune-mediated (CD8^+^ T and other immunocytes) and immune-independent (such as direct metabolic rewiring) mechanisms. These plausible mechanisms about BHB’s effect will be validated in the future studies.

From a clinical translation perspective, 1,3-butanediol supplement offers significant advantages over classical ketogenic diets for PDAC patients. The ketogenic diet requires extreme macronutrient restriction and carries risks of exacerbating cancer-related cachexia, a condition affecting ~80% of PDAC patients. Moreover, many PDAC patients have concurrent diabetes or exocrine pancreatic insufficiency, complicating adherence to strict dietary regimens. In contrast, 1,3-butanediol achieves therapeutic ketosis (~1.0–1.5 mM BHB, Fig. 1F) as a simple dietary supplement without caloric restriction. Critically, in healthy volunteers 1,3-butanediol supplement reached circulating BHB at ~1.0–1.5 mM and promoted metabolic fitness of peripheral T cells [13], prompting clinical trials testing 1,3-butanediol supplement for B-cell NHL CAR T-cell therapy (NCT06610344) and familial adenomatous polyposis (NCT06578637).

While encouraging, we acknowledge that our study was focused on a single syngeneic mouse model and did not directly quantify ketone utilization by cancer cells or intratumoral immune subsets using flux analysis, nor did we define BHB-driven signaling pathways. Metabolic tracing and deeper mechanistic investigation are warranted in future experiments to fully dissect the complexity of dietary interventions and safely translate the findings into clinical benefits. Lastly, ketogenic interventions have shown therapeutic synergy in other KRAS-driven PDAC models. In KPC mice, ketogenic diets have shown complementarity with gemcitabine and chemotherapy [15,16], suggesting that the general principle of metabolic adjuvancy extends beyond the iKRAS model. Beyond PDAC, we have previously shown that 1,3-butanediol enhances ICB in prostate cancer models [8], indicating that BHB-mediated immune augmentation is not PDAC-specific. Nonetheless, the metabolic heterogeneity of individual tumors, including varying degrees of glycolytic dependence, oxidative capacity, and baseline immune infiltration, may influence the magnitude of ketogenic benefit, warranting validation in additional KRAS-mutant models and genetically diverse settings.

## Supplementary Material

1

2

3

## Figures and Tables

**Fig. 1.**
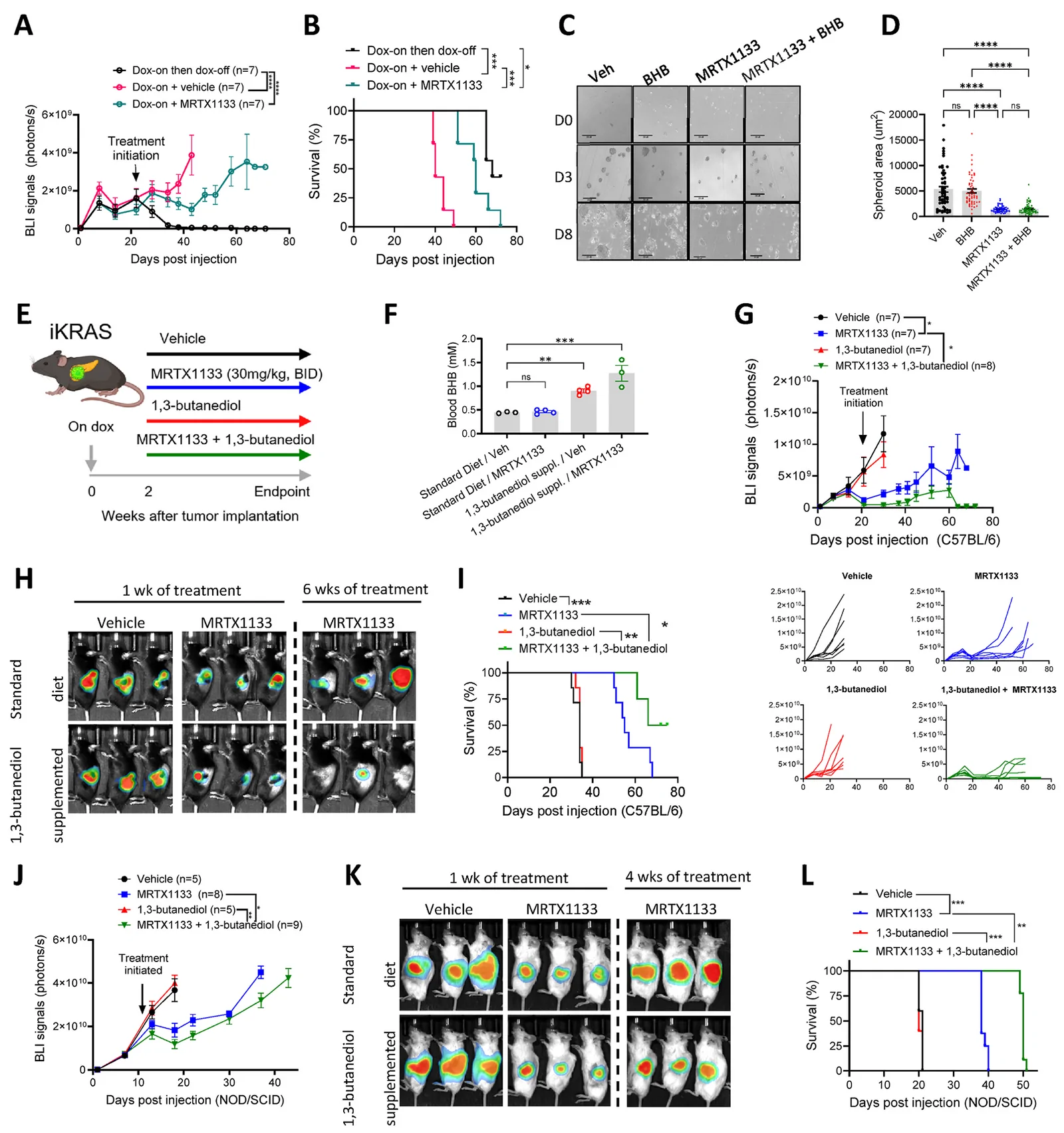
Ketogenic supplementation enhances the therapeutic efficacy of MRTX1133 in a murine pancreatic cancer model. **(A)** iKRAS pancreatic tumor progression measured by bioluminescence imaging (BLI). C57BL/6 mice started on-dox at day 0, and the arrow indicated the time switching to dox-off (black) or starting treatment with vehicle (red) or MRTX1133 (green). Group size (n) was shown. **(B)** Survival curves of tumor-bearing mice treated as in (A). **(C**–**D)** Representative images and area quantification of iKRAS spheroids cultured with doxycycline (2 μg/ml) and treated with vehicle (DMSO), β-hydroxybutyrate (BHB, 2.5 mM), MRTX1133 (100 nM), or the combination over 8 days. Scale bar 250 μm. **(E)** Experimental design to test MRTX1133 and dietary supplement with 1,3-butanediol, singly or together, in iKRAS pancreatic cancer model. **(F)** Serum BHB levels from mice, measured with ELISA. **(G)** iKRAS pancreatic tumor progression measured by BLI and shown as averaged or individual curves. C57BL/6 mice started on-dox at day 0, and arrow indicated the time to initiate treatment with vehicle, MRTX1133 (30 mg/kg, BID, intraperitoneal), 1,3-butanediol supplement in diet, or both. Group size (n) was shown. **(H)** Representative BLI images of tumor-bearing mice from each group at week 1 and week 6 after treatment initiation. Note that mice in vehicle and 1,3-butanediol groups arrived at endpoint before 6 weeks. **(I)** Survival curves of tumor-bearing mice treated as in (G). **(J-L**) BLI curves, representative BLI images, and survival curves for NOD/SCID mice treated similarly as in (G). In A, D, F, G and J, data represent mean ± SEM. *P < 0.05, **P < 0.01, ***P < 0.001, ****P < 0.0001, ns, not significant, based on log-rank test in B, I and L; two-way ANOVA with Tukey’s correction in A, D, G, J; two-way ANOVA with Dunnett’s correction in F. (For interpretation of the references to color in this figure legend, the reader is referred to the Web version of this article.)

**Fig. 2.**
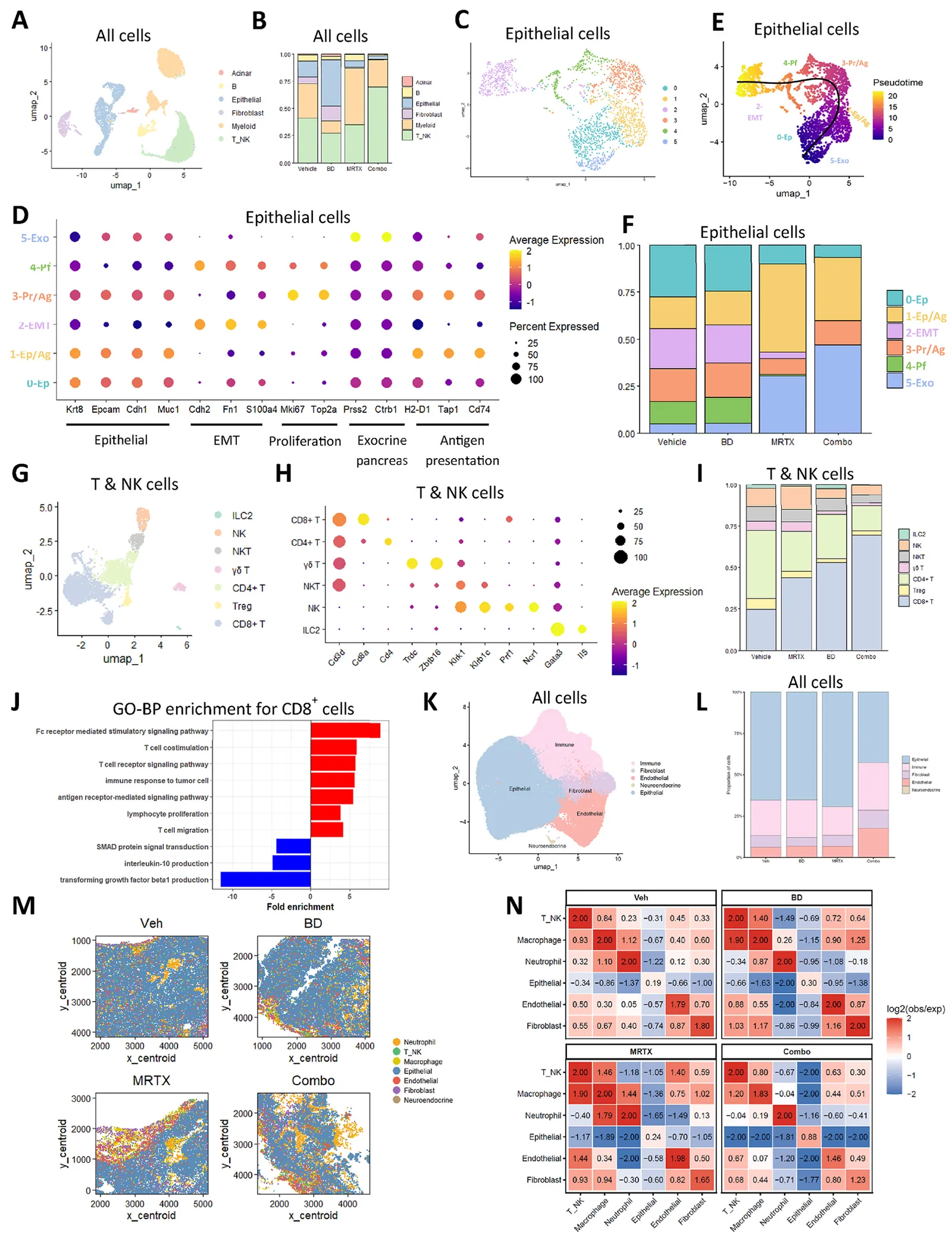
scRNA-seq and spatial transcriptomics reveal therapy–associated remodeling of cancer and immune compartments. **(A)** UMAP visualization of scRNA-seq profiles from orthotopic iKRAS pancreatic tumors (n = 2 per group) collected after 1 week of treatment, colored by major cell classes (acinar, epithelial, fibroblast, myeloid, B, and T/NK). **(B)** Relative abundance of major cell classes across treatment groups – Vehicle, 1,3-butanediol diet (BD), MRTX1133 (MRTX), and combination (Combo). **(C)** UMAP of epithelial cells taken from the total scRNA-seq data, colored by clusters (0–5)**. (D)** Dot plot showing expression of epithelial markers (*Krt8, Epcam, Cdh1, Muc1*), epithelial–mesenchymal transition (*Cdh2, Fn1, S100a4*), proliferation (*Mki67, Top2a*), exocrine pancreas (*Prss2, Ctrb1*), and antigen presentation (*H2-D1, Tap1, Cd74*) across epithelial clusters. Color indicates average scaled expression; dot size indicates percent of cells expressing each gene. **(E)** Pseudotime analysis of epithelial clusters using Slingshot, overlaid on the epithelial UMAP in (C). **(F)** Relative abundance of epithelial clusters across four treatment groups. **(G)** UMAP of T/NK compartment taken from the total scRNA-seq data and re-clustered into refined populations**. (H)** Dot plot showing expression of marker genes supporting lymphocyte subset annotations. **(I)** Relative abundance of the refined lymphocytes across treatment conditions. **(J)** Enrichment of GO biological pathways (BP) for differentially expressed genes in CD8^+^ T cells between Combo condition and the average of all other treatment conditions. Positive values indicate enrichment in Combo; negative values indicate the opposite. **(K)** UMAP of spatially profiled cells (n = 1 per group) from Xenium data, colored by major cell types (epithelial, immune, fibroblast, endothelial, neuroendocrine). **(L)** Relative abundance of major cell classes across treatment conditions. **(M)** Representative spatial maps of segmented cells showing the distribution of refined cell types within tumor sections from each treatment group. Axes show spatial coordinates in micrometers. **(N)** Heatmap showing neighborhood analysis within each treatment group. Values represent log2(observed/expected) ratios for spatial proximity between cell type pairs, using a radius-based neighborhood definition (radius = 20 μm). (For interpretation of the references to color in this figure legend, the reader is referred to the Web version of this article.)

## Appendix A. Supplementary data

Supplementary data to this article can be found online at https://doi.org/10.1016/j.canlet.2026.218490.
