# Supplementary material for: Ketogenic supplementation enhances KRASG12D inhibitor efficacy in pancreatic ductal adenocarcinoma
Source: Cancer Lett. Author manuscript; Available in PMC 2026 Nov 11. (PMC13752827; doi:10.1016/j.canlet.2026.218490)
Supplement: Supplementary file 1 [file NIHMS2221922-supplement-3.pdf]

Completed

Complete with Docusign: authorship-change-request-form.pdf

From: Xin Lu | Envelope ID

Download

Recipients (5) Details

Signing Order Show recipient details

✓	Xuemin Lu<br>xlu1@nd.edu	Signed 3/17/2026 | 10:59:40 am<br>Signed in location
✓	Loan Duong<br>lduong@nd.edu	Signed 3/17/2026 | 11:38:40 am<br>Signed in location
✓	Jun Wan<br>junwan@iu.edu	Signed 3/17/2026 | 12:23:38 pm<br>Signed in location
✓	Melissa L. Fishel<br>mfishel@iu.edu	Signed 3/17/2026 | 12:37:53 pm<br>Signed in location
✓	Anita A. Turk<br>aaturk@iu.edu	Signed 3/17/2026 | 04:21:13 pm

Documents

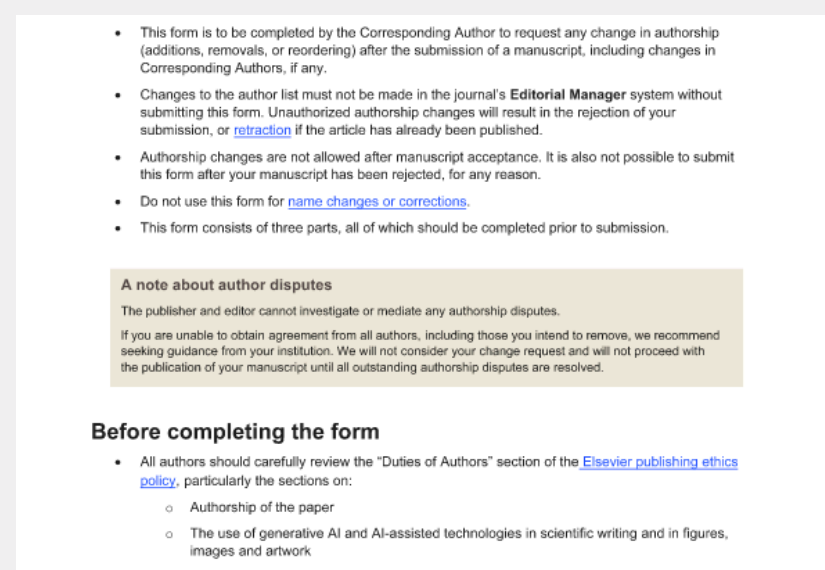

- This form is to be completed by the Corresponding Author to request any change in authorship (additions, removals, or reordering) after the submission of a manuscript, including changes in Corresponding Authors, if any.
- Changes to the author list must not be made in the journal's **Editorial Manager** system without submitting this form. Unauthorized authorship changes will result in the rejection of your submission, or retraction if the article has already been published.
- Authorship changes are not allowed after manuscript acceptance. It is also not possible to submit this form after your manuscript has been rejected, for any reason.
- Do not use this form for name changes or corrections.
- This form consists of three parts, all of which should be completed prior to submission.

A note about author disputes

The publisher and editor cannot investigate or mediate any authorship disputes.

If you are unable to obtain agreement from all authors, including those you intend to remove, we recommend seeking guidance from your institution. We will not consider your change request and will not proceed with the publication of your manuscript until all outstanding authorship disputes are resolved.

Before completing the form

- All authors should carefully review the "Duties of Authors" section of the Elsevier publishing ethics policy, particularly the sections on:
  - Authorship of the paper
  - The use of generative AI and AI-assisted technologies in scientific writing and in figures, images and artwork
- Please also carefully review the submission journal's "Guide for authors" (this might also be

authorship-change-request-...

7 pages

Subject
Complete with Docusign: authorship-change-request-form.pdf

Document
authorship-change-request-form.pdf

Document Id
a9a08d8f-4634-8991-80fd-f0497043e7ce

Recipients
Anita A. Turk, Jun Wan, Loan Duong, Melissa L. Fishel, Xuemin Lu

Date Sent
3/17/2026 | 10:58:23 am

Date Created
3/17/2026 | 10:50:43 am

Status Date
3/17/2026 | 04:21:13 pm

Location
Online

Holder
Xin Lu

Time Zone

Time	User	Action	Activity	Status
3/17/2026	10:50:43 am	Xin Lu	Registered	The envelope was created by Xin Lu	Created
3/17/2026	10:58:23 am	Xin Lu	Sent Invitations	Xin Lu sent an invitation to Anita A. Turk [aaturk@iu.edu]	Sent
3/17/2026	10:58:24 am	Xin Lu	Sent Invitations	Xin Lu sent an invitation to Jun Wan [junwan@iu.edu]	Sent
3/17/2026	10:58:24 am	Xin Lu	Sent Invitations	Xin Lu sent an invitation to Loan Duong [lduong@nd.edu]	Sent
3/17/2026	10:58:24 am	Xin Lu	Sent Invitations	Xin Lu sent an invitation to Melissa L. Fishel [mfishel@iu.edu]	Sent
3/17/2026	10:58:24 am	Xin Lu	Sent Invitations	Xin Lu sent an invitation to Xuemin Lu [xlu1@nd.edu]	Sent
3/17/2026	10:59:23 am	Xuemin Lu	Opened	Xuemin Lu opened the envelope [documents:(authorship-change-request-form.pdf)]	Sent
3/17/2026	10:59:31 am	Xuemin Lu	Viewed	Xuemin Lu viewed the envelope [documents:(authorship-change-request-form.pdf)]	Sent
3/17/2026	10:59:40 am	Xuemin Lu	Signed	Xuemin Lu signed the envelope	Sent
3/17/2026	11:37:41 am	Loan Duong	Opened	Loan Duong opened the envelope [documents:(authorship-change-request-form.pdf)]	Sent

Language	IP	Source
English (us)	129.74.47.247	api
English (us)	129.74.47.247	api
English (us)	129.74.47.247	api
English (us)	129.74.47.247	api
English (us)	129.74.47.247	api
English (us)	129.74.47.247	api
English (us)	129.74.34.16	web
En	129.74.34.16	web
En	129.74.34.16	web
English (us)	2600:1702:7f30:9430:15b2:4f91:57d2:6ef3	web

Time	User	Action	Activity	Status
3/17/2026 | 11:37:47 am	Loan Duong	Viewed	Loan Duong viewed the envelope [documents:(authorship-change-request-form.pdf)]	Sent
3/17/2026 | 11:38:40 am	Loan Duong	Signed	Loan Duong signed the envelope on mobile	Sent
3/17/2026 | 11:46:40 am	Xin Lu	Opened	Xin Lu opened the envelope [documents:(authorship-change-request-form.pdf)]	Sent
3/17/2026 | 11:46:44 am	Xin Lu	Viewed	Xin Lu viewed the envelope [documents:(authorship-change-request-form.pdf)]	Sent
3/17/2026 | 12:22:58 pm	Jun Wan	Opened	Jun Wan opened the envelope [documents:(authorship-change-request-form.pdf)]	Sent
3/17/2026 | 12:23:07 pm	Jun Wan	Viewed	Jun Wan viewed the envelope [documents:(authorship-change-request-form.pdf)]	Sent
3/17/2026 | 12:23:30 pm	Jun Wan	Opened	Jun Wan opened the envelope [documents:(authorship-change-request-form.pdf)]	Sent
3/17/2026 | 12:23:38 pm	Jun Wan	Signed	Jun Wan signed the envelope	Sent
3/17/2026 | 12:25:37 pm	Xin Lu	Opened	Xin Lu opened the envelope [documents:(authorship-change-request-form.pdf)]	Sent
3/17/2026 | 12:25:39 pm	Xin Lu	Viewed	Xin Lu viewed the envelope [documents:(authorship-change-request-form.pdf)]	Sent

Language	IP	Source
En	2600:1702:7f30:9430:15b2:4f91:57d2:6ef3	web
En	2600:1702:7f30:9430:15b2:4f91:57d2:6ef3	web
English (us)	129.74.47.247	web
En	129.74.47.247	web
English (us)	71.46.91.128	web
En	71.46.91.128	web
English (us)	72.153.153.38	web
En	71.46.91.128	web
English (us)	129.74.103.29	web
En	129.74.103.29	web

Time	User	Action	Activity	Status
3/17/2026 | 12:32:49 pm	Jun Wan	Opened	Jun Wan opened the envelope [documents:(authorship-change-request-form.pdf)]	Sent
3/17/2026 | 12:32:51 pm	Jun Wan	Viewed	Jun Wan viewed the envelope [documents:(authorship-change-request-form.pdf)]	Sent
3/17/2026 | 12:37:12 pm	Melissa L. Fishel	Opened	Melissa L. Fishel opened the envelope [documents:(authorship-change-request-form.pdf)]	Sent
3/17/2026 | 12:37:20 pm	Melissa L. Fishel	Viewed	Melissa L. Fishel viewed the envelope [documents:(authorship-change-request-form.pdf)]	Sent
3/17/2026 | 12:37:34 pm	Melissa L. Fishel	Opened	Melissa L. Fishel opened the envelope [documents:(authorship-change-request-form.pdf)]	Sent
3/17/2026 | 12:37:53 pm	Melissa L. Fishel	Signed	Melissa L. Fishel signed the envelope	Sent
3/17/2026 | 01:41:34 pm	Loan Duong	Opened	Loan Duong opened the envelope [documents:(authorship-change-request-form.pdf)]	Sent
3/17/2026 | 01:41:37 pm	Loan Duong	Viewed	Loan Duong viewed the envelope [documents:(authorship-change-request-form.pdf)]	Sent
3/17/2026 | 02:00:09 pm	Xin Lu	Opened	Xin Lu opened the envelope [documents:(authorship-change-request-form.pdf)]	Sent
3/17/2026 | 02:00:13 pm	Xin Lu	Viewed	Xin Lu viewed the envelope [documents:(authorship-change-request-form.pdf)]	Sent

Language	IP	Source
English (us)	71.46.91.128	web
En	71.46.91.128	web
English (us)	149.166.41.151	web
En	149.166.41.151	web
English (us)	72.152.84.228	web
En	149.166.41.151	web
English (us)	66.254.228.218	web
En	66.254.228.218	web
English (us)	129.74.47.247	web
En	129.74.47.247	web

Time	User	Action	Activity	Status
3/17/2026 | 04:20:38 pm	Anita A. Turk	Opened	Anita A. Turk opened the envelope [documents:(authorship-change-request-form.pdf)]	Sent
3/17/2026 | 04:20:48 pm	Anita A. Turk	Viewed	Anita A. Turk viewed the envelope [documents:(authorship-change-request-form.pdf)]	Delivered
3/17/2026 | 04:20:49 pm	Anita A. Turk	Opened	Anita A. Turk opened the envelope [documents:(authorship-change-request-form.pdf)]	Delivered
3/17/2026 | 04:21:13 pm	Anita A. Turk	Signed	Anita A. Turk signed the envelope on mobile	Completed
3/17/2026 | 04:21:16 pm	Xin Lu	Printable Copy Attached to Email	Xin Lu was sent the document (authorship-change-request-form.pdf) attached to the completed email	Completed
3/17/2026 | 04:21:17 pm	Anita A. Turk	Printable Copy Attached to Email	Anita A. Turk was sent the document (authorship-change-request-form.pdf) attached to the completed email	Completed
3/17/2026 | 04:21:18 pm	Jun Wan	Printable Copy Attached to Email	Jun Wan was sent the document (authorship-change-request-form.pdf) attached to the completed email	Completed
3/17/2026 | 04:21:18 pm	Loan Duong	Printable Copy Attached to Email	Loan Duong was sent the document (authorship-change-request-form.pdf) attached to the completed email	Completed
3/17/2026 | 04:21:19 pm	Melissa L. Fishel	Printable Copy Attached to Email	Melissa L. Fishel was sent the document (authorship-change-request-form.pdf) attached to the completed email	Completed
3/17/2026 | 04:21:20 pm	Xuemin Lu	Printable Copy Attached to Email	Xuemin Lu was sent the document (authorship-change-request-form.pdf) attached to the completed email	Completed

Language	IP	Source
English (us)	2a09:bac3:7ca6:8c::e:365	web
En	2a09:bac3:7ca6:8c::e:365	web
English (us)	74.179.68.93	web
En	2a09:bac3:7ca6:8c::e:365	web
Na	2a09:bac3:7ca6:8c::e:365	web
Na	2a09:bac3:7ca6:8c::e:365	web
Na	2a09:bac3:7ca6:8c::e:365	web
Na	2a09:bac3:7ca6:8c::e:365	web
Na	2a09:bac3:7ca6:8c::e:365	web
Na	2a09:bac3:7ca6:8c::e:365	web

Time	User	Action	Activity	Status
3/17/2026 | 04:21:53 pm	Loan Duong	Opened	Loan Duong opened the envelope [documents:(authorship-change-request-form.pdf)]	Completed
3/17/2026 | 04:21:58 pm	Loan Duong	Viewed	Loan Duong viewed the envelope [documents:(authorship-change-request-form.pdf)]	Completed
3/17/2026 | 04:24:47 pm	Xin Lu	Opened	Xin Lu opened the envelope [documents:(authorship-change-request-form.pdf)]	Completed
3/17/2026 | 04:24:52 pm	Xin Lu	Viewed	Xin Lu viewed the envelope [documents:(authorship-change-request-form.pdf)]	Completed
3/17/2026 | 04:25:11 pm	Jun Wan	Opened	Jun Wan opened the envelope [documents:(authorship-change-request-form.pdf)]	Completed
3/17/2026 | 04:25:13 pm	Xin Lu	Printable Copy Delivered	Xin Lu received a printable copy of the envelope	Completed
3/17/2026 | 04:25:15 pm	Jun Wan	Viewed	Jun Wan viewed the envelope [documents:(authorship-change-request-form.pdf)]	Completed
3/17/2026 | 04:25:35 pm	Jun Wan	Opened	Jun Wan opened the envelope [documents:(authorship-change-request-form.pdf)]	Completed
3/17/2026 | 04:25:44 pm	Jun Wan	Viewed	Jun Wan viewed the envelope [documents:(authorship-change-request-form.pdf)]	Completed
3/17/2026 | 05:55:03 pm	Loan Duong	Opened	Loan Duong opened the envelope [documents:(authorship-change-request-form.pdf)]	Completed

Language	IP	Source
English (us)	66.254.228.218	web
En	66.254.228.218	web
English (us)	129.74.47.247	web
En	129.74.47.247	web
English (us)	71.46.91.128	web
En	129.74.47.247	web
En	71.46.91.128	web
English (us)	72.152.84.108	web
En	72.152.84.108	web
English (us)	66.254.228.218	web

Time	User	Action	Activity	Status
3/17/2026 | 05:55:06 pm	Loan Duong	Viewed	Loan Duong viewed the envelope [documents:(authorship-change-request-form.pdf)]	Completed

Language	IP	Source
En	66.254.228.218	web

docusign Home Agreements Templates Reports Admin 13 Day

Completed

Complete with Docusign: Docusign_authorship-change-50% Completed.pdf

From: Xin Lu | Envelope ID

Download

Recipients (4) Details

Signing Order Show recipient details

Nipuni Barupala<br>nbarupal@nd.edu	Signed 3/17/2026 | 04:31:14 pm	Signed in location
Yujing Qin<br>yqin5@nd.edu	Signed 3/17/2026 | 04:33:20 pm	Signed in location
Julim Lee<br>jlee2345@nd.edu	Signed 3/17/2026 | 05:09:56 pm	Signed in location
Xin Lu<br>xlu@nd.edu	Signed 3/17/2026 | 06:24:28 pm	Signed in location

Documents

Docusign_authorship-change-50% Completed.pdf

Docusign_authorship-chang...
7 pages

Subject
Complete with Docusign: Docusign_authorship-change-50% Completed.pdf

Document
Docusign_authorship-change-50% Completed.pdf

Document Id
d7ca455c-3ab8-8bcd-82fc-848e0d9c44db

Recipients
Julim Lee, Nipuni Barupala, Xin Lu, Yujing Qin

Date Sent
3/17/2026 | 04:28:37 pm

Date Created
3/17/2026 | 04:26:35 pm

Status Date
3/17/2026 | 06:24:28 pm

Location
Online

Holder
Xin Lu

Time Zone

Time	User	Action	Activity	Status
3/17/2026 | 04:26:35 pm	Xin Lu	Registered	The envelope was created by Xin Lu	Created
3/17/2026 | 04:28:37 pm	Xin Lu	Sent Invitations	Xin Lu sent an invitation to Julim Lee [jlee2345@nd.edu]	Sent
3/17/2026 | 04:28:37 pm	Xin Lu	Sent Invitations	Xin Lu sent an invitation to Nipuni Barupala [nbarupal@nd.edu]	Sent
3/17/2026 | 04:28:37 pm	Xin Lu	Sent Invitations	Xin Lu sent an invitation to Yujing Qin [yqin5@nd.edu]	Sent
3/17/2026 | 04:28:37 pm	Xin Lu	Sent Invitations	Xin Lu sent an invitation to Xin Lu [xlu@nd.edu]	Sent
3/17/2026 | 04:29:17 pm	Yujing Qin	Opened	Yujing Qin opened the envelope [documents:(Docusign_authorship-change-50% Completed.pdf)]	Sent
3/17/2026 | 04:29:28 pm	Yujing Qin	Viewed	Yujing Qin viewed the envelope [documents:(Docusign_authorship-change-50% Completed.pdf)]	Sent
3/17/2026 | 04:29:35 pm	Nipuni Barupala	Opened	Nipuni Barupala opened the envelope [documents:(Docusign_authorship-change-50% Completed.pdf)]	Sent
3/17/2026 | 04:29:46 pm	Nipuni Barupala	Viewed	Nipuni Barupala viewed the envelope [documents:(Docusign_authorship-change-50% Completed.pdf)]	Sent
3/17/2026 | 04:31:14 pm	Nipuni Barupala	Signed	Nipuni Barupala signed the envelope on mobile	Sent

Language	IP	Source
English (us)	129.74.47.247	api
English (us)	129.74.47.247	api
English (us)	129.74.47.247	api
English (us)	129.74.47.247	api
English (us)	129.74.47.247	api
English (us)	66.254.228.59	web
En	66.254.228.59	web
English (us)	129.74.34.199	web
En	129.74.34.199	web
En	129.74.34.199	web

Time	User	Action	Activity	Status
3/17/2026 | 04:31:25 pm	Nipuni Barupala	Printable Copy Delivered	Nipuni Barupala received a printable copy of the envelope	Sent
3/17/2026 | 04:33:20 pm	Yujing Qin	Signed	Yujing Qin signed the envelope	Sent
3/17/2026 | 04:36:48 pm	yqin5	Printable Copy Delivered	yqin5 received a printable copy of the envelope	Sent
3/17/2026 | 05:08:13 pm	Julim Lee	Opened	Julim Lee opened the envelope [documents:(Docusign_authorship-change-50% Completed.pdf)]	Sent
3/17/2026 | 05:08:45 pm	Julim Lee	Viewed	Julim Lee viewed the envelope [documents:(Docusign_authorship-change-50% Completed.pdf)]	Sent
3/17/2026 | 05:09:56 pm	Julim Lee	Signed	Julim Lee signed the envelope	Sent
3/17/2026 | 06:17:55 pm	Xin Lu	Opened	Xin Lu opened the envelope [documents:(Docusign_authorship-change-50% Completed.pdf)]	Sent
3/17/2026 | 06:18:03 pm	Xin Lu	Viewed	Xin Lu viewed the envelope [documents:(Docusign_authorship-change-50% Completed.pdf)]	Delivered
3/17/2026 | 06:18:43 pm	Xin Lu	Printable Copy Delivered	Xin Lu received a printable copy of the envelope	Delivered
3/17/2026 | 06:23:13 pm	Xin Lu	Opened	Xin Lu opened the envelope [documents:(Docusign_authorship-change-50% Completed.pdf)]	Delivered

Language	IP	Source
En	129.74.34.199	web
En	66.254.228.59	web
English (us)	66.254.228.59	api
한국어	66.254.228.168	web
Ko	66.254.228.168	web
Ko	66.254.228.168	web
English (us)	98.52.243.163	web
En	98.52.243.163	web
En	98.52.243.163	web
English (us)	2601:245:c300:890:447:a10:7238:af36	web

Time	User	Action	Activity	Status
3/17/2026 | 06:23:16 pm	Xin Lu	Viewed	Xin Lu viewed the envelope [documents:(Docusign_authorship-change-50% Completed.pdf)]	Delivered
3/17/2026 | 06:24:28 pm	Xin Lu	Signed	Xin Lu signed the envelope	Completed
3/17/2026 | 06:24:30 pm	Xin Lu	Printable Copy Attached to Email	Xin Lu was sent the document (Docusign_authorship-change-50% Completed.pdf) attached to the completed email	Completed
3/17/2026 | 06:24:31 pm	Julim Lee	Printable Copy Attached to Email	Julim Lee was sent the document (Docusign_authorship-change-50% Completed.pdf) attached to the completed email	Completed
3/17/2026 | 06:24:31 pm	Nipuni Barupala	Printable Copy Attached to Email	Nipuni Barupala was sent the document (Docusign_authorship-change-50% Completed.pdf) attached to the completed email	Completed
3/17/2026 | 06:24:32 pm	Yujing Qin	Printable Copy Attached to Email	Yujing Qin was sent the document (Docusign_authorship-change-50% Completed.pdf) attached to the completed email	Completed
3/17/2026 | 06:24:54 pm	Xin Lu	Printable Copy Delivered	Xin Lu received a printable copy of the envelope	Completed

Language	IP	Source
En	2601:245:c300:890:447:a10:7238:af36	web
En	2601:245:c300:890:447:a10:7238:af36	web
Na	2601:245:c300:890:447:a10:7238:af36	web
Na	2601:245:c300:890:447:a10:7238:af36	web
Na	2601:245:c300:890:447:a10:7238:af36	web
Na	2601:245:c300:890:447:a10:7238:af36	web
English (us)	2601:245:c300:890:447:a10:7238:af36	api
