# Supplementary material for: Ketogenic supplementation enhances KRASG12D inhibitor efficacy in pancreatic ductal adenocarcinoma
Source: Cancer Lett. Author manuscript; Available in PMC 2026 Nov 11. (PMC13752827; doi:10.1016/j.canlet.2026.218490)
Supplement: Supplementary file 2 [file NIHMS2221922-supplement-1.docx]

Supplementary Data for

**Ketogenic supplementation enhances KRAS^G12D^ inhibitor efficacy in pancreatic ductal adenocarcinoma**

**Duong et al.**

**This file includes:**

- Materials and Methods
- Supplementary Figures S1 and S2

**Materials and Methods**

## 1.1. Cell lines and *in vitro* treatment

iKRAS cell line AK14838, syngeneic to the C57BL/6 background, was developed from the *P48-Cre; R26-rtTa-IRES-EGFP*; *tetO-LSL-Kras^G12D/+^; Trp53^L/+^* genetically engineered mouse model by Haoqiang Ying’s group as previously described [9] and shared with us. Cells were maintained in culture in RPMI1640 medium (HyClone, SH30027) supplemented with 10% heat-inactivated fetal bovine serum (HyClone, SH3039603), 1% penicillin-streptomycin (HyClone, SV30010) and 2 µg/ml doxycycline (VWR 103516-792). All the cultures were maintained at 37 °C in a humidified incubator with 5% CO2. The mycoplasma-free status was verified using the Mycoplasma Detection Kit (ABM, G238). Cells were treated with MRTX1133 (Mirati Therapeutics) and/or β-hydroxybutyrate (Cayman Chemical, 14148) at the indicated concentrations.

## 1.2. Immunoblotting

Cells were lysed in RIPA buffer supplemented with protease inhibitors (MedChemExpress, #HY-K0011) and phosphatase inhibitors (Bimake, B15002). The western blot procedure has been described previously [8]. The primary antibodies used include β-actin (Santa Cruz Biotechnology, sc-47778), Vinculin (Sigma-Aldrich, #05-386), phospho-MEK (Cell Signaling Technology, # 9121), MEK (Cell Signaling Technology, #9122), phosphor-ERK (Cell Signaling Technology, #4370), ERK (Cell Signaling Technology, #4695), KRAS (Sigma-Aldrich, # OP24), cleaved caspase 3 (Cell Signaling Technology, #9661). Secondary antibodies included HRP-linked anti-rabbit IgG (Cell Signaling Technology, #7074) and HRP-linked anti-mouse IgG (Cell Signaling Technology, #7076).

## 1.3. Orthotopic tumors in mice

All animal work performed in this study was approved by the Institutional Animal Care and Use Committee (IACUC) at University of Notre Dame. All animals were maintained under pathogen-free conditions and cared for per the International Association for Assessment and Accreditation of Laboratory Animal Care policies and certification. C57BL/6J mice were purchased from the Jackson Laboratory (#000664). NOD-SCID mice (NOD.CB17-*Prkdc^scid^*/NCrCrl) were purchased from Charles River Laboratories (#394).

For orthotopic modeling, C57BL/6 or NOD/SCID female mice aged 6-10 weeks were anesthetized using isoflurane. After making an incision in the left abdomen, the spleen and pancreas were gently pulled out. 5 x 10^5^ iKRAS cells (stably labeled with luciferase-zsGreen (Addgene, #39196) in 1:1 cold PBS: Matrigel (Corning, # 354230) were slowly injected into the pancreas. Analgesic was administered before surgery. The mice were maintained on doxycycline chow (Bio-Serv S3888) ad libitum after the procedure before changing to standard chow (Teklad, 2918) or drug treatment as indicated. Tumor burden was monitored longitudinally by bioluminescence imaging (BLI) on the Spectral Ami HT imager following anesthetization and intravenous administration of D-luciferin. Endpoint was determined based on signs of lethargy, reduced mobility, and morbidity.

## 1.4. Therapeutic and dietary interventions

For 1,3-butanediol supplementation, we followed our previous report [8] and prepared the diet by mixing 80 mL of 1,3-butanediol (Sigma-Aldrich, 309443), 120 mL of water, 198 g of doxycycline chow, and 2 g of saccharin (Sigma-Aldrich, 109185). For MRTX1133 treatment, the vehicle was prepared by dissolving captisol powder (MedChemExpress, HY-17031) in 50 mmol/L citrate buffer (pH 5.0) to prepare 10% (w/v) captisol solution. MRTX1133 powder was dissolved in 10% captisol solution. For MRTX1133 treatment, mice were intraperitoneally injected with MRTX1133 at 30 mg/kg body weight twice daily.

## 1.5. Single-cell RNA sequencing and data analysis

Tumors were harvested from mice (n=2 per group) and immediately transferred to a 10-cm culture dish containing digestion medium consisting of 1 mg/mL collagenase/dispase prepared in RPMI1640 supplemented with 10% fetal bovine serum. Tumor tissues were mechanically minced using sterile razor blades and incubated in digestion medium at 37 °C with rotation for 1 hour. Following digestion, samples were pelleted, resuspended in RPMI1640 with 10% FBS, and mechanically dissociated by passage through 18-gauge and 20-gauge needles. The resulting suspension was filtered through a 70-µm cell strainer, pelleted, and red blood cells were removed by incubation with 1x RBC lysis buffer on ice. Following this, the cells were pelleted and subject to dead-cell removal using the EasySep Annexin V Dead Cell Removal Kit (STEMCELL Technologies) according to the manufacturer’s instructions, with two sequential depletion steps performed to enrich viable cells. Following dead-cell removal, cells were processed for sample multiplexing with TotalSeq-A0303 (BioLegend, 155805), TotalSeq-A0304 (BioLegend, 155807), TotalSeq-A0305 (BioLegend, 155809), TotalSeq-A0306 (BioLegend, 155811), TotalSeq-A0307 (BioLegend,), and TotalSeq-A0308 (BioLegend, 155815). At this step, the samples were transferred to Genomics and Bioinformatics Core Facility at University of Notre Dame, where cells were counted, evenly combined, and loaded onto the Chromium Controller (10X Genomics). The samples were then processed following the manuals of the Chromium Single Cell 3′ Reagent Kits. Single-cell RNA-seq libraries were generated using the 10x Genomics Chromium platform and sequenced on an Illumina NovaSeq X Plus instrument.

Raw sequencing data were processed using the Cell Ranger pipeline (10x Genomics) for barcode processing, alignment to the mouse reference genome, and UMI counting. Samples were multiplexed using BioLegend TotalSeq hashtag oligonucleotide antibodies and demultiplexed before downstream analysis. All downstream analyses were performed in R using the Seurat package. Cells were filtered based on standard quality-control metrics, including the number of detected genes, total UMI counts, and mitochondrial transcript percentage, resulting in a final dataset of 13,966 cells across four treatment groups (Vehicle, BD, MRTX, and combination therapy). Gene expression values were log-normalized and highly variable genes were identified for downstream analysis. Data were scaled prior to dimensionality reduction using principal component analysis (PCA), and the first 10 principal components were used to generate Uniform Manifold Approximation and Projection (UMAP) embeddings. Cell clustering was performed by constructing a shared nearest-neighbor graph followed by graph-based Louvain clustering. Cell identities were assigned based on canonical marker gene expression and reference-based annotation, followed by manual consolidation into major cell populations. Differential gene expression analyses were performed using the Wilcoxon rank-sum test implemented in Seurat.

For Gene Ontology Biological Process (GO-BP) analysis, first, differentially expressed genes (DEGs) were identified by comparing the specified cell type in the Combo condition against the same cell type pooled from all other treatment conditions using Seurat's FindMarkers function (Wilcoxon rank-sum test; log2 fold change threshold > 0.25, adjusted p-value < 0.05). Next, GO-BP over-representation analysis was performed separately on upregulated DEGs and downregulated DEGs using the clusterProfiler R package, with significance threshold set at adjusted p-value < 0.05.

## 1.6. Spatial transcriptomics and data analysis

Spatial transcriptomic profiling was performed on pancreatic tumor tissues (one representative tumor per group) using the Xenium In Situ platform (10x Genomics). Tumor tissues were formalin-fixed, paraffin-embedded (FFPE), sectioned at 5 µm thickness, and mounted onto Xenium-compatible slides. Spatial gene expression was measured using the Xenium Mouse Multi-Tissue panel supplemented with a custom Xenium add-on gene panel. In situ hybridization, signal amplification, decoding, and fluorescence imaging were performed on the Xenium Analyzer according to the manufacturer’s standard Xenium In Situ Gene Expression protocols.

Cell segmentation and transcript assignment were performed using the Xenium onboard analysis pipeline, which incorporates nuclear and cytoplasmic boundary information to assign detected transcripts to individual cells. Following initial processing, cell-level gene expression matrices and spatial metadata were imported into R for downstream analysis using the Seurat framework. Cells were filtered based on quality-control metrics. Due to the large number of cells recovered, cells were randomly downsampled and analyzed using normalized gene expression values for dimensionality reduction, clustering, and cell-type annotation based on canonical marker gene expression. Spatial visualization and region-of-interest analyses were performed using Xenium-derived cellular coordinates.

Cellular neighborhood enrichment analysis was performed to quantify spatial co-localization between cell types. For each condition, cell-type neighbor pairs were identified within a 30 µm radius using the RANN package in R. Neighborhood enrichment scores were calculated as log2(observed neighbor proportion / expected neighbor proportion), where observed proportions reflect the frequency of each cell type among a given cell's neighbors, and expected proportions are derived from the overall cell-type frequency within that condition.

## 1.7. Statistical analysis.

In vitro and in vivo experiments were performed three or more times, and conclusions were drawn only when the results were reproducible. One representative result among the replicates is shown in the figures. For non-omics data, statistical analyses were performed using GraphPad Prism. All data are presented as mean ± SEM (standard error of the mean) with all data points shown. We followed this workflow for statistical testing: Shapiro-Wilk test was performed to assess for normality of data distribution: (i) in case of normality, when only two conditions were to test, we performed unpaired t-test; when more than two conditions were to compare, we performed a parametric one-way or two-way ANOVA followed by post hoc test with recommended correction for multiple comparisons to assess the significance among pairs of conditions. (ii) in case of non-normality, when only two conditions were to test, we performed a Mann-Whitney U test; when more than two conditions were to compare, we performed a non-parametric one-way ANOVA followed by recommended test to assess the significance among pairs of conditions. All statistical tests reported are two-sided unless otherwise indicated. For survival data, log rank test was used.  Sample sizes, error bars, P values, and statistical methods are noted in the figure legends. Statistical significance was defined as P value <0.05.

## 1.8. Data availability

Raw and processed count matrices for scRNA-seq and ST were deposited in Gene Expression Omnibus (GEO) under accession GSE320285. All other raw data and materials generated in this study are available upon reasonable request to the corresponding authors.

**Supplementary Figures**

# Supplementary Figure S1. MRTX1133 suppresses KRAS–MAPK signaling in iKRAS cells but demonstrated limited long-term tumor control.

**(A)** Immunoblot showing doxycycline (dox, 2 µg/ml)-dependent induction of KRAS^G12D^ and MAPK signaling (phospho-MEK, phospho-ERK) in iKRAS cell line. **(B)** Immunoblot showing dose-dependent suppression of MAPK signaling by MRTX1133 in iKRAS cells under dox-on conditions. Cells were treated with the indicated concentrations of MRTX1133 for 6 hours. **(C)** Immunoblot showing time-dependent suppression of MAPK signaling following MRTX1133 (100 nM) treatment for the indicated durations in iKRAS cells under dox-on condition. The 12+12h time point indicates cells treated for 12 hours followed by media replacement with fresh MRTX1133 for an additional 12 hours, representing 24 hours of total drug exposure with re-dosing at the midpoint. **(D)** Longitudinal tumor progression measured by bioluminescence imaging (BLI) in NOD/SCID mice bearing orthotopic luciferase-labeled iKRAS tumors. Total photon emission was quantified over time following treatment initiation (arrow) in mice maintained on dox (dox-on) and treated with vehicle or MRTX1133, or switched to dox-off to extinguish KRAS^G12D^ expression. Data are shown as mean ± SEM. **(E)** Kaplan–Meier survival analysis of tumor-bearing NOD/SCID mice treated as in (D). Statistical significance was determined using a log-rank (Mantel–Cox) test (**P < 0.01, ****P < 0.0001). **(F)** Immunoblot of apoptosis marker cleaved caspase-3 in iKRAS spheroids treated for 24 hours with β-hydroxybutyrate (BHB, 2.5 mM), MRTX1133 (100 nM), or the combination under dox-on condition. **(G)** Individual BLI curves tracking iKRAS pancreatic tumor progression in NOD/SCID mice treated with vehicle, MRTX1133 (30 mg/kg, BID, intraperitoneal), 1,3-butanediol supplement in diet, or both. The average curves, group size (n), representative BLI images, and survival curves were shown in Figure 1J-L. *P < 0.05, **P < 0.01, ***P < 0.001, ****P < 0.0001, two-way ANOVA with Tukey’s correction in D.

**H**

**I**

**J**

**K**

**L**

Myeloid cells

GO-BP enrichment for neutrophil cells

**C**

All cells

**A**

TME Cells

Myeloid cells

All cells

Immune cells

Immune cells

Myeloid cells

TCR signaling complex

Effector cytotoxicity

Co-stimulation

Master TF

Tissue Infiltration

CD8^+^ T cells

**D**

**B**

CD8^+^ T cells

Checkpoint receptors

**E**

Exhaustion and suppression

CD8^+^ T cells

**F**

**G**

Vehicle

1,3-butanediol

1,3-butanediol

+ MRTX1133

MRTX1133

Neutrophil

T_NK

Macrophage

Epithelial

Endothelial

Fibroblast

Neuroendocrine

**M**

# Supplementary Figure S2. Treatment reshapes the myeloid and immune compartments in orthotopic iKRAS pancreatic tumors.

**(A)** Dot plot visualization showing expression of marker genes of major cell classes in scRNA-seq data. Color indicates average scaled expression; dot size indicates percent of cells expressing each gene. **(B)** Relative abundance of tumor microenvironment cells (T_NK, Myeloid, Fibroblast, B cells) across treatment groups. **(C)** Dot plot visualization of CD8⁺ T cell activation gene signatures across treatment groups (Vehicle, MRTX, BD, Combo). Genes are grouped by functional category: TCR signaling complex, effector cytotoxicity, co-stimulation, master transcription factor (TF), and tissue infiltration. **(D)** Dot plot visualization of CD8^+^ T cell exhaustion and suppression markers across treatment groups. **(E)** Dot plot visualization of checkpoint receptor expression in CD8^+^ T cells across treatment groups. **(F)** UMAP visualization of myeloid cells from tumor scRNA-seq data, colored by myeloid subsets (neutrophils, monocytes/macrophages, and dendritic cells). **(G)** Dot plot showing expression of marker genes supporting myeloid subset annotations. Color indicates average scaled expression; dot size indicates percent of cells expressing each gene. **(H)** Relative abundance of myeloid subsets across treatment groups (Vehicle, 1,3-BD, MRTX1133, and combination), plotted as proportions within the myeloid compartment. **(I)** GO Biological Process over-representation analysis (ORA) of differentially expressed genes in neutrophil cells comparing Combo versus all other treatment conditions. Pathways with positive values (red) were upregulated (UP) in the combination condition, whereas pathways with negative values (blue) were downregulated (DOWN) in the Combo condition. **(J)** Dot plot showing expression of marker genes used to assign major cell classes in Xenium spatial transcriptomics data. Color indicates average scaled expression; dot size indicates percentage of cells expressing each gene. **(K)** UMAP visualization of spatially profiled immune cells from Xenium data, colored by resolved immune subsets (T/NK, macrophages, and neutrophils). **(L)** Dot plot showing expression of canonical marker genes supporting Xenium immune subset annotations (color indicates average scaled expression; dot size indicates percent of cells expressing each gene). **(M)** Individual spatial plots by treatment condition and annotated cell type as shown in Figure 2M.
